# Decreased but persistent epigenetic age acceleration is associated with changes in T-cell subsets after initiation of highly active antiretroviral therapy in persons living with HIV

**DOI:** 10.3389/fbinf.2024.1356509

**Published:** 2024-05-24

**Authors:** Mary E. Sehl, Elizabeth Crabb Breen, Roger Shih, Fengxue Li, Joshua Zhang, Peter Langfelder, Steve Horvath, Jay H. Bream, Priya Duggal, Jeremy Martinson, Steven M. Wolinsky, Otoniel Martinez-Maza, Christina M. Ramirez, Beth D. Jamieson

**Affiliations:** ^1^ Division of Hematology-Oncology, Department of Medicine, David Geffen School of Medicine at UCLA, University of California Los Angeles, Los Angeles, CA, United States; ^2^ Department of Computational Medicine, David Geffen School of Medicine at UCLA, University of California Los Angeles, Los Angeles, CA, United States; ^3^ Department of Psychiatry and Biobehavioral Sciences, Cousins Center for Psychoneuroimmunology, David Geffen School of Medicine at UCLA, University of California, Los Angeles, Los Angeles, CA, United States; ^4^ Department of Biostatistics, Fielding School of Public Health, University of California, Los Angeles, Los Angeles, CA, United States; ^5^ Department of Human Genetics, David Geffen School of Medicine at UCLA, University of California, Los Angeles, Los Angeles, CA, United States; ^6^ Center for Neurobehavioral Genetics, Jane and Terry Semel Institute for Neuroscience and Human Behavior, University of California Los Angeles, Los Angeles, CA, United States; ^7^ Department of Psychiatry and Biobehavioral Sciences, David Geffen School of Medicine at UCLA, University of California Los Angeles, Los Angeles, CA, United States; ^8^ Altos Labs, San Diego Institute of Science, San Diego, CA, United States; ^9^ Department of Molecular Microbiology and Immunology, Johns Hopkins Bloomberg School of Public Health, Immunology Training Program, Johns Hopkins School of Medicine, Baltimore, MD, United States; ^10^ Department of Epidemiology, Johns Hopkins Bloomberg School of Public Health, Baltimore, MD, United States; ^11^ Department of Infectious Diseases and Microbiology, Graduate School of Public Health, University of Pittsburgh, Pittsburgh, PA, United States; ^12^ Department of Medicine, Northwestern University Feinberg School of Medicine, Chicago, IL, United States; ^13^ Departments of Obstetrics and Gynecology and Microbiology, Immunology and Molecular Genetics, David Geffen School of Medicine at UCLA, University of California, Los Angeles, CA, United States

**Keywords:** human immunodeficiency virus, aging, DNA methylation, epigenetic clock, antiretroviral therapy

## Abstract

**Introduction:**

Persons living with HIV (PLWH) experience the early onset of age-related illnesses, even in the setting of successful human immunodeficiency virus (HIV) suppression with highly active antiretroviral therapy (HAART). HIV infection is associated with accelerated epigenetic aging as measured using DNA methylation (DNAm)-based estimates of biological age and of telomere length (TL).

**Methods:**

DNAm levels (Infinium MethylationEPIC BeadChip) from peripheral blood mononuclear cells from 200 PLWH and 199 HIV-seronegative (SN) participants matched on chronologic age, hepatitis C virus, and time intervals were used to calculate epigenetic age acceleration, expressed as age-adjusted acceleration residuals from 4 epigenetic clocks [Horvath’s pan-tissue age acceleration residual (AAR), extrinsic epigenetic age acceleration (EEAA), phenotypic epigenetic age acceleration (PEAA), and grim epigenetic age acceleration (GEAA)] plus age-adjusted DNAm-based TL (aaDNAmTL). Epigenetic age acceleration was compared for PLWH and SN participants at two visits: up to 1.5 years prior and 2–3 years after HAART (or equivalent visits). Flow cytometry was performed in PLWH and SN participants at both visits to evaluate T-cell subsets.

**Results:**

Epigenetic age acceleration in PLWH decreased after the initiation of HAART but remained greater post-HAART than that in age-matched SN participants, with differences in medians of 6.6, 9.1, and 7.7 years for AAR, EEAA, and PEAA, respectively, and 0.39 units of aaDNAmTL shortening (all *p* < 0.001). Cumulative HIV viral load after HAART initiation was associated with some epigenetic acceleration (EEAA, PEAA, and aaDNAmTL), but even PLWH with undetectable HIV post-HAART showed persistent epigenetic age acceleration compared to SN participants (*p* < 0.001). AAR, EEAA, and aaDNAmTL showed significant associations with total, naïve, and senescent CD8 T-cell counts; the total CD4 T-cell counts were associated with AAR, EEAA, and PEAA (*p* = 0.04 to <0.001). In an epigenome-wide analysis using weighted gene co-methylation network analyses, 11 modules demonstrated significant DNAm differences pre- to post-HAART initiation. Of these, nine were previously identified as significantly different from pre- to post-HIV infection but in the opposite direction.

**Discussion:**

In this large longitudinal study, we demonstrated that, although the magnitude of the difference decreases with HAART is associated with the cumulative viral load, PLWH are persistently epigenetically older than age-matched SN participants even after the successful initiation of HAART, and these changes are associated with changes in T-cell subsets.

## Introduction

Human immunodeficiency virus (HIV) infection accelerates epigenetic aging as measured using DNA methylation-based estimates of biological age (Rickabaugh et al., 2015; Gross et al., 2016; Sehl et al., 2020; Sehl et al., 2021; Breen et al., 2022; Breen et al., 2023), as well as DNA methylation-based estimates of telomere length (DNAmTL) that are reflective of cell replicative history (Sehl et al., 2021; Breen et al., 2022; Breen et al., 2023). We recently demonstrated that epigenetic age acceleration occurs at the time of initial HIV infection as measured using several epigenetic clocks and DNAmTL. We also found that counts and proportions of naïve and senescent lymphocyte subsets are associated with the degree of epigenetic acceleration (Breen et al., 2022; Breen et al., 2023). Although recent studies have demonstrated that epigenetic age acceleration persists after the initiation of antiretroviral therapy, with a diminished degree of age acceleration in the setting of successful suppression (Sehl et al., 2020; Esteban-Cantos et al., 2021; Esteban-Cantos et al., 2022; Schoepf et al., 2023), no prior studies have examined the relationship between cell composition, as directly measured by flow cytometry, with alterations in epigenetic age acceleration in response to highly active antiretroviral therapy (HAART).

In this study, we examined 5 measures of epigenetic aging and directly measured T-cell subset counts in 200 persons living with HIV (PLWH) within 1.5 years prior to HAART initiation and again in the same persons 2–3 years post-HAART initiation. The five epigenetic measures we examined include age-adjusted acceleration residuals for four epigenetic clocks, Horvath age acceleration residual (AAR), extrinsic epigenetic age acceleration (EEAA), phenotypic epigenetic age acceleration (PEAA), and grim epigenetic age acceleration (GEAA), and age-adjusted DNA methylation telomere length (aaDNAmTL). In brief, these measures are based on the weighted averages of methylation levels at 353, 71, 513, 1,030, and 140 CpGs. In brief, while AAR is a pan-tissue clock, EEAA exhibits negative and positive correlations with naïve and late-differentiated/senescent cytotoxic T lymphocytes, respectively. PEAA is highly correlated with age-related phenotypes, and GEAA is strongly predictive of the lifespan. Finally, aaDNAmTL is a measure of cell replicative history. For comparison, we evaluated the same epigenetic aging measures over matched time intervals in persistently HIV-seronegative (SN) persons matched on chronological age and hepatitis C virus status. Both PLWH and matched HIV negative controls had extensive information on factors that could potentially contribute to epigenetic aging, and within PLWH, we examined whether HIV suppression and improved CD4 counts after HAART initiation were associated with the degree of epigenetic acceleration. In addition to the five epigenetic measures, we examined methylome-wide changes that were associated with HAART initiation in PLWH. Because of the wealth of clinical and flow cytometry data available at each visit for both PLWH and matched controls, we were able to test the hypothesis of whether plasma HIV viral load (VL), CD4 T-cell counts, and cell composition changes accompany the degree of persistent epigenetic age acceleration that remains after the initiation of HAART.

## Materials and methods

### Human subjects

Samples were selected from participants of the Multicenter AIDS Cohort Study (MACS), now part of the MACS/WIHS Combined Cohort Study (MWCCS). The MACS is an ongoing prospective study of the natural and treated history of HIV infection in men who have sex with men (Kaslow et al., 1987). Samples for the current substudy of the initiation of HAART were selected whenever possible from MACS participants whose samples contributed to a large biomarker study, which has been described elsewhere (Wada et al., 2015). The MWCCS complies with all relevant ethical regulations, including obtaining informed consent for the research from all study participants. The MACS/MWCCS substudy described herein was given exempt status by the University of California, Los Angeles Medical Institutional Review Board IRB#15001179.

A total of 200 PLWH who initiated HAART after entry into the MACS were selected and matched to 199 persistently HIV-SN men. Selection criteria are described below in *Methods*. Clinical and demographic information about all participants is given in Table 1.

**TABLE 1:** Characteristics of persons living with HIV (PLWH) and matched HIV-seronegative (SN) participants from the Multicenter AIDS Cohort Study (MACS) in the study sample.

	PLWH^a^		SN^b^	*p*-value^c^
**Non-white race, n (%)**	29 (14.5)		73 (36.5)	<0.001
**Hispanic ethnicity, n (%)**	16 (8.0)		16 (8.0)	1
**≥1 year college education, n (%)**	180 (90.0)		164 (82.0)	0.03
**Visit 1 to visit 2, years, mean (SD)**	2.9 (0.4)		2.7 (0.8)	0.01

At each visit	PLWH		SN	
	Visit 1	Visit 2	Visit 1	Visit 2
**Age, years, mean (SD), [range]**	44.0 (7.8) [24.1–72.4]	46.8 (7.9) [27.0–75.5]	43.8 (8.0) [22.3–71.1]	46.5 (8.1) [24.7–73.8]
**Hepatitis C virus RNA-positive, n (%)**	8 (4.0)	10 (5.0)	8 (4.0)	10 (5.0)
**Hepatitis B virus surface antigen-positive, n (%)**	4 (2.0), n = 199	5 (2.5), n = 199	5 (2.5), n = 200	5 (2.5), n = 200
**Body mass index, kg/m** ^**2**^ **, mean (SD)**	25.5 (4.0), n = 187	25.4 (3.7), n = 186	26.1 (4.8), n = 191	26.6 (4.9), n = 189
**Smoking, cumulative pack years, mean (SD)**	13.6 (19.6), n = 196	14.3 (20.6), n = 197	10.7 (15.1), n = 195	11.2 (15.8), n = 195
**Absolute CD4 T-cell count, cells/mm** ^**3**^ **, mean (SD)**	414 (235), n = 198	555 (277), n = 199	997 (323), n = 193	969 (299), n = 196
**Plasma HIV viral load, copies/mL, mean (SD), median [range]** ^**d**^	80,374 (190,028)	9,443 (57,313)	n/a	n/a
	21,818 [50–1,733,258], n = 189	50 [20–775,459], n = 199		
**Estimated time since HIV infection, years, mean (SD)**	9.3 (4.3), n = 173	12.1 (4.4), n = 173	n/a	n/a

a: n = 200 PLWH at pre-HAART (visit 1) and post-HAART (visit 2), unless indicated otherwise.

b: n = 400 unique visits for 199 unique SN individuals; one seronegative participant matched to two different PLWH but at non-overlapping MACS visits.

c: *p*-values from Student’s t-tests for continuous variables and chi-squared tests for count data (*p*-values bold if <0.05).

d: At post-HAART (visit 2), PLWH had median viral loads of 50 copies/mL, with 62.5% PLWH at 50 copies/mL or below.

The original predetermined sample size calculation was as follows: a sample size of 200 achieves 80% power to detect an R-squared of 0.10 attributed to 3 independent variables using an F-test with a significance level (alpha) of 0.05 and adjusted for an additional 3 confounding variables.

### Definition and initiation of HAART

The initiation of HAART was determined according to the absence and presence of the reported use of antiretroviral medications at each MACS visit, confirmed by medical record review, that met the definition of HAART previously described for the large biomarker study from which all of the PLWH in the current methylation study were drawn (Wada et al., 2015; Castillo-Mancilla et al., 2016; Castillo-Mancilla et al., 2020). HAART was defined using the 2008 definition that was in use at the time of the biomarker study—at least three antiretroviral therapies, i.e., two nucleoside reverse transcriptase inhibitors (NRTIs) plus either an unboosted protease inhibitor (PI) or a boosted PI or a non-nucleoside reverse transcriptase inhibitor (NNRTI) (Castillo-Mancilla et al., 2020). Supplementary Table S1 summarizes information about what types of antiretroviral medication, including currently taking and ever taken, were used by the 200 PLWH in our study. PLWH sample selection, as described below, was anchored on the first MACS visit at which HAART was reported, i.e., all prior MACS visits were pre-HAART.

### Participant selection and samples

Viably frozen peripheral blood mononuclear cells (PBMCs) were obtained from the national repository of the MACS/MWCCS. The MACS visits typically occur at 6-month intervals, clinical and questionnaire data are collected, and peripheral blood samples are processed and frozen.

**FIGURE 1:**
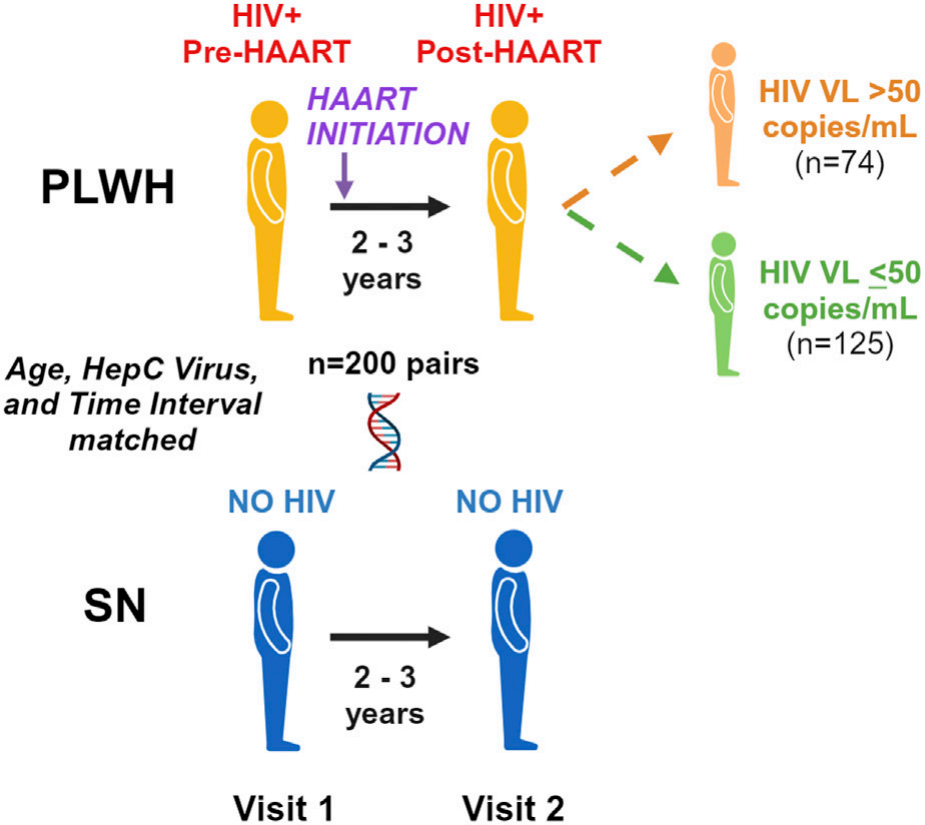
Pre/post-highly active antiretroviral therapy (HAART) study design. We examine accelerated aging pre-HAART and 2–3 years after the initiation of HAART in persons living with HIV (PLWH) and age-matched seronegative (SN) men over the same time period. We further examine differences between those PLWH with detected (>50 copies/mL) and undetected (≤ 50 copies/mL) plasma HIV viral loads at the post-HAART visit.

Figure 1 illustrates our pre- and post-HAART study designs. A total of 200 PLWH were selected who had PBMC samples available in the repository from two time periods: (1) up to 1.5 years prior to the first MACS visit at which HAART was reported (pre-HAART, visit 1) and (2) up to 2.5 years after the first HAART visit (post-HAART, visit 2). If multiple PBMC samples were available after HAART initiation, the post-HAART visit closest to 3 years after the pre-HAART visit was selected. Men with a documented new HIV infection after entry into the MACS were selected first (n = 117), followed by men who were HIV-seropositive upon entry into the MACS (n = 83), for a total of 200 unique PLWH and 400 PBMC samples.

Persistently HIV uninfected SN controls were then selected among the MACS participants from the larger biomarker study when possible, or from among persistently SN MACS participants at large, matched to each PLWH participant. SN controls were matched by age (±2 years) and hepatitis C virus (HCV) status (HCV RNA-positive/negative) at both visits, as well as by the availability of PBMCs at two visits with a comparable time interval between the visits (±0.75 years, equivalent to pre- and post-HAART visits). In total, 199 matched SN controls were identified, with 1 SN control participant matched at 4 different visits to 2 PLWH participants due to age and HCV criteria, yielding 400 unique SN PBMC samples.

### Participant demographics and characteristics

Absolute CD4 T-cell counts, hepatitis B virus (HBV) status, plasma HIV VL (in PLWH), and other demographic and clinical data were available from the MWCCS database and are summarized in Table 1. Where data are missing from the 400 person-visits from 200 PLWH at two time points or 400 unique person-visits for 199 SN participants at two time points (1 SN control matched to 2 different PLWH but at non-overlapping visits), the exact “N” is shown in Table 1. The date of HIV infection for the 117 PLWH participants who became HIV-infected after entry into the MACS was estimated as the midpoint between the last HIV-seronegative and/or HIV VL undetectable MACS visit and the first MACS visit with either HIV-positive serostatus or detectable HIV VL, whichever came first. For 56 PLWH, who were HIV-seropositive at entry into the MACS when it began in 1983–1984, the date of their first MACS visit was used as the estimated date of infection as they should not have been infected for very long before enrollment, given the history of the HIV/AIDS epidemic. A total of 27 PLWH were HIV-seropositive at MACS entry but entered later in the history of the study and, so, were considered missing for the estimated date of HIV infection. The date of HAART initiation was estimated as the midpoint between the last MACS visit at which no HAART was reported and the first MACS visit at which HAART was reported. For PLWH, visits with undetectable VL, a value equal to the lower limit of detection of the VL assay was assigned (<400 copies/mL, Roche Amplicor second-generation assay, Roche Molecular Systems, Branchburg, NJ, United States; <50 copies/mL, ultra-sensitive Roche Amplicor assay). HBV status at each visit was categorized as HBV surface antigen (HBsAg) positive or negative.

Cumulative HIV VL (CVL) was calculated across two time periods for each PLWH: (1) CVL at the post-HAART visit since the estimated date of HAART initiation (CVL-H) and (2) CVL at the post-HAART visit since the estimated date of HIV infection (CVL-I). CVL, expressed as viremia copy-years, was calculated using the method proposed by Wang et al. (2018)

Eleven PLWH were missing a viral load value at the pre-HAART visit from which the samples were selected. For eight of those participants, the viral load from the following visit, which fell within 6 months of the pre-HAART visit but before the estimated date of HAART initiation, was used. Because those criteria were not met for the remaining three participants, the viral load from the closest preceding visit, but within 6 months of the pre-HAART visit, was substituted for two of the three participants. The remaining individuals did not meet any of those criteria, so no CVL was calculated. Only one participant had a missing viral load at the post-HAART visit, so the viral load from the following visit, which was within 6 months of the post-HAART visit, was substituted.

### Thawing and viability of frozen samples

Frozen PBMC vials were removed from liquid nitrogen storage tanks, thawed, counted, and viability assessed as described elsewhere (Breen et al., 2022; Breen et al., 2023); the mean viability of PBMCs was 89.6% across all vials, all visits. Thawed PBMCs were divided for DNA extraction and flow cytometry.

### Genomic DNA isolation and quantification

A washed and dried pellet of 1.0 × 10^6^ viable PBMCs per sample was stored in a −80-C freezer until genomic DNA isolation. DNA was extracted from the frozen dry PBMC pellets as described elsewhere (Breen et al., 2022; Breen et al., 2023). DNA concentration was determined using a NanoDrop One system (Thermo Fisher) using the dsDNA setting and automatic measurements generated from wavelengths of 220–340 nm. Genomic DNA samples were then stored in −80-C freezers until being plated for methylation analysis.

### DNA methylation arrays

Blinded matched sets of genomic DNA samples were created by the investigators, with each set containing samples from matched PLWH and SN participants at all visits. The sets were placed on an Infinium MethylationEPIC BeadChip (Illumina, San Diego, CA), and the methylation status at more than 850,000 potential methylation sites (CpGs) was determined by the UCLA Neuroscience Genomics Core (https://www.semel.ucla.edu/ungc), as previously described (Breen et al., 2022; Breen et al., 2023). DNA methylation levels (beta values) were determined by calculating the intensity of the methylated and unmethylated sites as the ratio of fluorescent signals, yielding beta values that range from 0 (completely unmethylated) to 1 (completely methylated). Quantile normalization was applied to the raw data to detect and remove outliers and make data comparable to the training data of the epigenetic clocks and consistent with previous analyses (Sehl et al., 2020).

### Epigenetic age acceleration measures

Five measures of epigenetic age acceleration were estimated for each of the 800 PBMC samples in total, representing samples from all participants at both visits, using the online epigenetic clock software application (http://dnamage.genetics.ucla.edu). Each of these DNA methylation-based estimates was calculated using methylation beta values obtained from the Infinium MethylationEPIC BeadChip on all samples at the same time without linkage to the HIV serostatus group. There were no adjustments for multiple comparisons as each epigenetic measure was developed taking this into account. Features of each clock examined are provided in our previous report (Breen et al., 2022; Breen et al., 2023). In brief, AAR is based on the DNAm age estimated from 353 CpGs of Horvath’s original epigenetic clock (Horvath, 2013), which is then regressed on chronological age. AAR captures epigenetic age acceleration (i.e., older epigenetic or biological age than chronological age), is valid for a wide range of tissue types, and is accelerated in disease states. EEAA is based on 71 CpGs of Hannum et al. (2013) and was constructed to be positively correlated with senescent T lymphocytes and negatively correlated with naive T lymphocytes (Hannum et al., 2013; Chen et al., 2016). This measure captures both intrinsic methylation changes and extrinsic blood cell composition changes. Second-generation clocks, including phenotypic age and grim age, were examined as they are much stronger predictors of mortality. PEAA, based on 513 CpGs, was developed by regressing a phenotypic measure of mortality risk on CpGs (Levine et al., 2018). GEAA, based on 1,030 CpGs, was developed by regressing the time to death on DNAm-based surrogate biomarkers of smoking pack years and a selection of plasma proteins previously associated with mortality or morbidity (Lu et al., 2019a). Finally, a DNA methylation-based estimator of telomere length adjusted for chronological age (aaDNAmTL) was examined in our analyses to evaluate whether HIV infection causes the accelerated shortening of telomeres with increased age and/or rate of cellular replication (Lu et al., 2019b).

### Flow cytometry for T-cell subsets

Percentages of naïve (CD45RA^+^CCR7^+^), activated (HLA-DR^+^CD38^+^), and senescent (CD28^−^CD57^+^) T cells within the total CD4 (CD3^+^CD4^+^) and CD8 (CD3^+^CD8^+^) T cells of each PBMC sample were determined by multicolor flow cytometry (Brenchley et al., 2003; Mahnke et al., 2013). Depending on recovery and viability after thawing, 0.5 × 10^6^–1.0 × 10^6^ viable PBMCs per tube were stained for flow cytometry on the same day as they were thawed, with a total of three staining tubes per sample (naïve/senescent, activated, and isotype). Out of 800 thawed aliquots of PBMCs used in DNAm analyses in PLWH and SN groups at visits 1 and 2, 790 had sufficient viable PBMCs to stain for flow cytometry. PBMCs were surface-stained, acquired, and analyzed as previously described (Breen et al., 2022; Breen et al., 2023), using an Attune NxT Flow Cytometer (Thermo Fisher A29004 [blue/red/violet6/yellow]), yielding up to approximately 800,000 cell events for analysis. Naïve and senescent CD4 and CD8 T cells were stained and analyzed in one tube, and activated CD4 and CD8 T cells were stained and analyzed in a separate tube. Due to technical issues during flow cytometry acquisition, the activated cell tube, or both tubes, was/were unable to be acquired on a small number of PBMC samples at one or both visits.

### Frequencies of T-cell subsets within the live lymphocyte population

Percentages of total CD3 T cells, CD4 T cells, and CD8 T cells, and naïve, activated, and senescent CD4 and CD8 T cells among the total live lymphocytes in each aliquot of thawed viable PBMCs were calculated as previously described (Breen et al., 2022). Of 790 PBMC samples with sufficient viable cells to stain for flow cytometry, 27 samples were excluded due to coefficients of variation (CVs) > 10% on mean % CD3, and one each was excluded due to CVs >10% on mean % CD4 and % CD8.

### Absolute counts of T-cell subsets

Absolute total CD4 and total CD8 T-cell counts (cells/mm^3^) for almost all samples were available from the MACS/MWCCS database, which were determined by standardized protocols on the day each blood sample was originally obtained (Giorgi et al., 1990; Hultin et al., 2007). Flow cytometry percentages on thawed PBMCs were used in combination with absolute total CD4 and CD8 T-cell counts to calculate the absolute cell counts for naïve, activated, and senescent CD4 and CD8 T cells for each sample. As noted above, on a small number of PBMC samples at one or both visits, activated or both naive and activated T-cell data were unable to be acquired, or percentage data were excluded due to high sample variability. In addition, a small number of absolute CD4 and CD8 T-cell counts were missing from the MWCCS database, resulting in some variability in the number of PBMC samples for which percentages were available and absolute cell counts could be calculated.

### Statistical analyses

To examine the impact of HAART on the epigenetic age in PLWH, samples were used from two time points: one occurring pre-HAART initiation and the second occurring ∼2.5 years post-HAART initiation. However, it is not possible to determine exactly when the observed changes in epigenetic age occur. However, “epigenetic age acceleration” measures are compared, defined as the residual that results from the regression of the epigenetic age on chronological age, at both visits for the PLWH and SN groups. Because these measures are age-adjusted, they are technically termed “accelerations” in the epigenetic clock literature rather than elevations or advancements, even though the time course and rate of acceleration are unknown.

### Statistical analyses of epigenetic measures and T-cell subsets

No raw methylation data, calculated epigenetic clock, or estimated DNAmTL data were excluded from the analyses. HIV serostatus groups (PLWH and SN participants) were compared to each other (*t*-test, two-sided) on each age-adjusted epigenetic measure at visit 1 (pre-HAART vs. equivalent visit) and again at visit 2 (post-HAART in PLWH vs. time interval-matched visit in SN participants). Within-person changes in age-adjusted epigenetic measures (visit 2–visit 1) were calculated for each participant. PLWH and SN groups were each evaluated by paired t-tests (two-sided) for differences from zero. Similar analyses were performed on the absolute counts of T-cell subsets and percentages of T-cell subsets within live lymphocytes, comparing PLWH vs. SN participants at pre-HAART vs. equivalent visits (visit 1) and again at post-HAART vs. equivalent visits (visit 2; *t*-test, two-sided), as well as evaluating within-person changes for the absolute T-cell counts from visit 1 to visit 2 in PLWH and SN participants (paired t-tests, two-sided, for differences from zero). Pairwise correlation analyses were performed among the five epigenetic measures for PLWH and SN participants at visits 1 and 2, and Pearson’s correlation coefficients (rho) and *p*-values were reported.

At the post-HAART visit in the PLWH group, linear regression analyses were performed for each of the epigenetic measures and CVL-I and CVL-H. In all analyses where HIV VL was included, the viral load (copies/mL) was log10-transformed. For the analyses of CVL, regression coefficient estimates/t-values and *p*-values were reported.

Additional epigenetic analyses were performed with the PLWH group stratified based on VL at the post-HAART visit (visit 2) into detected (VL > 50 copies/mL, n = 74) and undetected (VL ≤ 50 copies/mL, n = 125); one PLWH participant had insufficient VL data to be included in these analyses. To evaluate epigenetic aging in the two PLWH groups, the mean of the detected group was compared at each visit to the mean of the matched SN controls for that group at the equivalent visit by paired t-tests and likewise for the undetected group and their matched SN participant. The means of the two PLWH groups were also compared to each other at each visit by paired t-tests.

Potential contributions of demographic covariates (study visit [visit 1 vs. 2], HIV group [PLWH vs. SN participant], interaction between study visit and the HIV group, race [non-white vs. white], current hepatitis B status [HBsAg negative vs. positive], body mass index [BMI], and tobacco smoking [cumulative pack years]) to the changes in AAR, EEAA, PEAA, and aaDNAmTL between the two visits (visit 1 and visit 2) were analyzed in linear mixed-effects models with all participants at both visits in the same model. Demographic covariates were determined *a priori* based on variables that were widely available (i.e., minimal missing data in the MACS database) and had the potential to affect aging. GEAA was not included in any of the mixed models since it did not show any significant change between visits in either PLWH or SN participants. Due to missing data for some demographic covariates, n = 391 samples were used for these mixed models.

Potential contributions of absolute counts of T-cell subsets (total CD4, total CD8, naïve CD4, naïve CD8, activated CD4, activated CD8, senescent CD4, and senescent CD8) to the changes in the four age-adjusted epigenetic measures between the two visits were analyzed in linear mixed-effects models with all participants in the same model. All absolute T-cell counts were natural log-transformed (ln cells/mm^3^) before inclusion into mixed models. Due to missing data for some flow cytometry variables, n = 388 samples were used for these mixed models. Six potential models were evaluated across the four epigenetic measures. Each model was constructed with a different combination of 3–5 T-cell subsets, based on the expected HIV pathogenesis and/or to minimize expected highly correlated subsets to reduce collinearity. We selected the model with the consensus best fit across the four epigenetic measures that were included in the models using the Akaike Information Criterion (AIC); this model included total CD4, total CD8, naïve CD8, activated CD8, and senescent CD8.

### Weighted gene correlation network analysis of genomic methylation data

Weighted gene correlation network analysis (WGCNA) (Langfelder and Horvath, 2008) was used to identify clusters of CpG DNA methylation sites that are correlated with each other across the genomic DNA (co-methylation modules) of the samples analyzed (all samples from all participants at both visits, n = 800 samples) using methylation levels measured at over 850,000 individual CpG methylation sites on the Infinium MethylationEPIC BeadChip. Missing values were imputed using k-nearest neighbor imputation implemented in the impute.knn function in R package impute (Troyanskaya et al., 2001). CpGs were then sorted by decreasing variance, and the top 400,000 CpGs were retained for WGCNA. Because network analysis on 400,000 CpGs in a single block is impractical, pre-clustering implemented in the WGCNA R package was used to split the CpGs into blocks of no more than 40,000 variables. We restricted our analysis to the top varying 400,000 CpGs, which represent approximately the top half of the overall CpGs (over 850,000), in order to optimize our ability to capture co-varying CpG information to identify WGCNA modules (which are typically found within the top 30% of the co-varying CpGs), while remaining within the range of computational feasibility (Langfelder and Horvath, 2008). We performed a “blockwise” (block by block) analysis as an approximation to single-block analysis. We chose a block size of 40,000 CpGs in order to optimize the block size within the constraints of computational feasibility. This approach accurately captures biological findings as in a single-block analysis because very large modules that are split into several modules will have highly correlated eigen-CpGs (Langfelder and Horvath, 2008). Network construction and module identification were then carried out in each block separately. Average linkage hierarchical clustering was performed using the topological overlap-based dissimilarity measure, and modules, defined as branches of the resulting clustering tree, were identified using the dynamic hybrid branch cutting approach implemented in dynamicTreeCut of the R package. A total of 67 co-methylation modules were identified using WGCNA, identical to those previously published by us in a study of HIV seroconversion (Breen et al., 2022; Breen et al., 2023). A representative methylation profile for each module, referred to as the module eigenvector, was defined as the first principal component of the module methylation matrix, and for each CpG within each module, the intramodular connectivity measure kME was calculated as follows:

$$kME_{i}^{I}=cor\left(x_{i},E^{l}\right),$$

where *x*_*i*_ is the methylation profile of the CpG labeled *i* and *E* ^*I*^ is the representative of module *I*. kME can be considered a continuous measure of module membership for each CpG (Horvath and Dong, 2008). When a CpG has a high kME for a given module (e.g., above a threshold value ≥ 0.85), it is considered a hub site.

Mean and standard deviation (SD) eigenvector methylation values for each module at each visit were calculated separately on PLWH (n = 200 individuals, 200 unique PBMC samples at each visit) and on SN participants (n = 199 individuals, 200 unique PBMC samples at each visit). Non-parametric group comparison tests (Kruskal–Wallis) were performed in each group, comparing the mean module eigenvector methylation value from pre-HAART or equivalent visit to post-HAART or equivalent visit. The level of significance for changes in the mean module eigenvector methylation values adjusting for multiple comparisons was *p* < 0.05/67 or <7.5 × 10^−4^. In PLWH, 11 out of 67 modules had a *p*-value < 7.5 × 10^−4^. None of the 67 modules showed significant differences between the two visits in the SN participants and were not analyzed further. For each of the 11 significant modules in PLWH, the direction and magnitude of methylation changes pre- to post-HAART were calculated as the mean visit 2 eigenvector minus the mean visit 1 eigenvector; positive numbers represent increases in methylation, while negative numbers represent decreases in methylation following the initiation of HAART.

Nine of the 11 HAART initiation-associated modules have been previously described, and detailed lists of all CpGs in each of those modules are given in the study by Breen et al. (2022) All of the CpGs from each of the two novel HAART initiation-associated modules are listed in Supplementary Table S6, which is a supplemental Excel file. The file lists, on a separate tab for each module, the CpG Illumina ID number for the unique site on the EPIC BeadChip, name(s) of the gene(s) that contain(s) the unique CpG site, and the kME value for each CpG.

### Pathways enrichment analyses of genomic methylation data

Enrichment analyses were performed using the Enrichr gene list enhancement tool (Kuleshov et al., 2016) to identify overrepresented biological pathways for those CpG sites with high connectivity (kME ≥ 0.85 from WGCNA) within each of the 11 HAART initiation-associated modules. Supplementary Table S7 (a supplemental Excel file) lists, on a separate tab for each module, enrichment terms (names of biological processes or pathways) identified in the Enrichr analysis, along with the overlap (number of genes identified in our analysis over the total number of genes in the literature for a given pathway), *p*-value (computed using Fisher’s exact test, assuming a binomial distribution and independence for probability of any gene belonging to any set), adjusted *p*-value (correction for all known genes in the set), odds ratio for each enrichment term [given by (a/b)/(c/d), where a = number of genes in the module that fall in the enrichment term, b = number of genes in the module that do not fall in the enrichment term, c = number of genes in the enrichment term that are not in the module, and d = 20,000–(a+b + c), where 20,000 is the total number of genes in the human genome], and combined score (the product of the log of the *p*-value from Fisher’s exact test with the z-score deviation from the expected rank for each term in each gene-set library) for each enrichment term identified for each module. Enrichment terms within each module are ordered based on the *p*-value, from most to least significant.

## Results

### Demographics

Table 1 shows the characteristics of the PLWH and persistent HIV-SN controls from the MACS, who were included in the current substudy, examining the effects of HAART initiation on epigenetic aging. Ages of the participants ranged from 22 to 74 years, with most of the participants being white, non-Hispanic, college-educated or beyond, and many had no history of smoking tobacco (N = 84 (42%) PLWH; N = 76 (38%) SN participants). No significant differences were observed between PLWH and matched SN participants in age, tobacco smoking pack years, BMI, active HBV infection, and hepatitis C infection. As previously reported in a related substudy (Breen et al., 2022; Breen et al., 2023), MACS participants evaluated early in the cohort study showed nearly universal seropositivity for cytomegalovirus (CMV, 97%–100%) prior to HIV infection, so CMV infection was not evaluated for the current substudy. A significantly lower proportion (14.5%) of non-white PLWH than for SN participants (36.5%) and a significantly higher percentage of PLWH (90%) who completed at least 1 year of college than SN participants (82%) were observed. By design, the time intervals between samples at visit 1, the pre-HAART (taking place up to 1.5 years prior to the first MACS visit at which HAART was reported) or equivalent visit, and visit 2, the post-HAART (taking place up to 2.5 years after the first HAART visit) or equivalent visit, were very similar in PLWH (mean 2.9 years, range 1.0–3.8) and matched SN participants (mean 2.7, range 0.6–4.5). As expected, the mean CD4 T-cell counts at both visits were significantly lower in PLWH than those in SN participants (Supplementary Table S3). In PLWH, at the pre-HAART visit, the median HIV viral load was 21,818 copies/mL (range 50–1,722,258 copies/mL). At the post-HAART visit, the majority (62.5%) of PLWH had a viral load of ≤ 50 copies/mL.

Epigenetic age acceleration decreases after the initiation of HAART but remains accelerated compared to matched seronegative controls.

**FIGURE 2:**
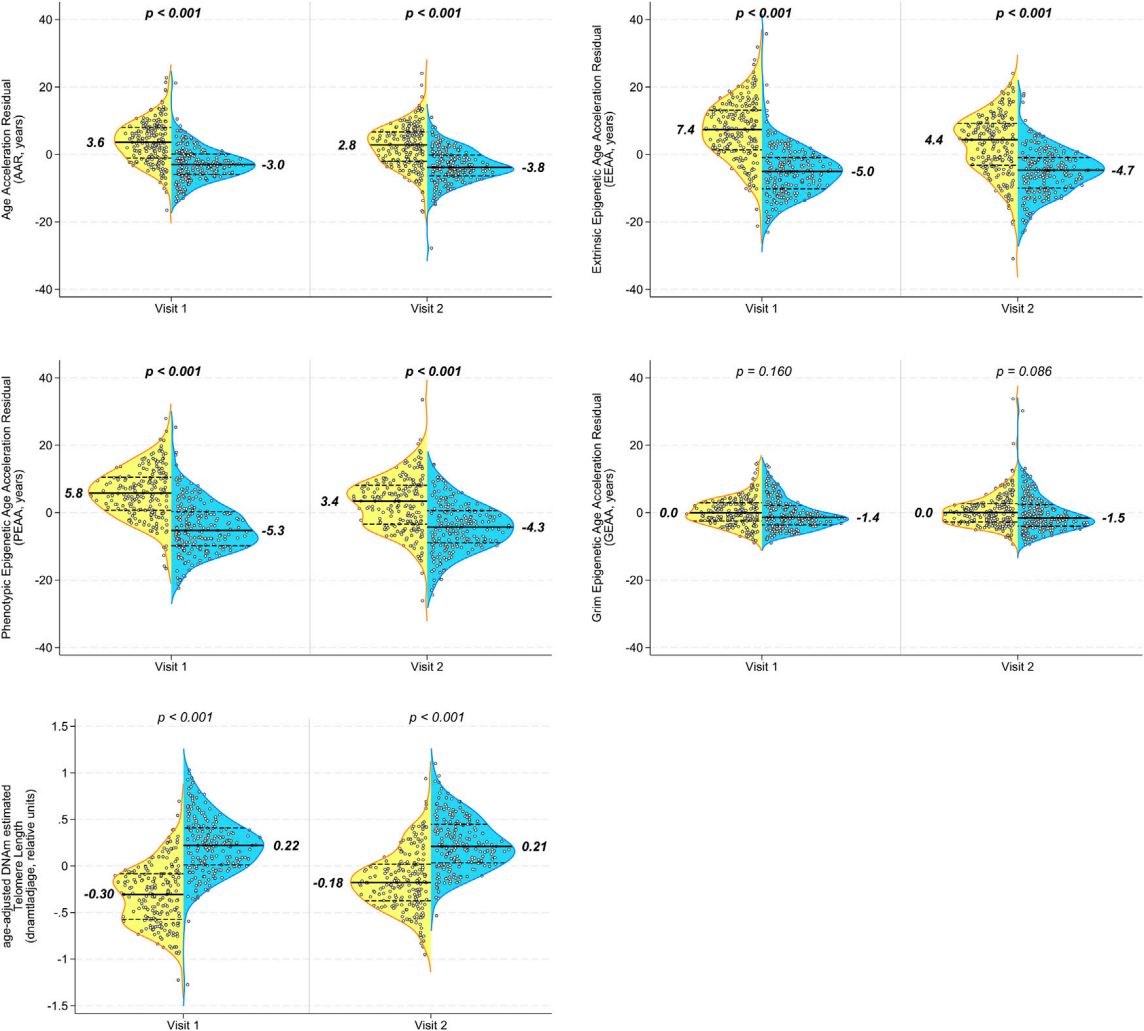
Multiple epigenetic measures in peripheral blood mononuclear cells (PBMCs) demonstrate persistent significant differences in biological aging between PLWH and age-matched SN men before and after the initiation of HAART. Longitudinal PBMC samples from men before (visit 1) and 2–3 years after (visit 2) the initiation of HAART, and from matched (chronological age, hepatitis C status, and time interval) SN men, were evaluated for biologic aging using five different age-adjusted epigenetic measures **(A)** Age acceleration residual (AAR), **(B)** extrinsic epigenetic age acceleration (EEAA), **(C)** phenotypic epigenetic age acceleration (PEAA), **(D)** grim epigenetic age acceleration (GEAA), and **(E)** age-adjusted DNA methylation-based estimate of telomere length (aaDNAmTL). The first four are epigenetic clocks that increase with aging, whereas the estimated TL shortens (decreases) with aging. Each panel shows violin plots for PLWH (left half, yellow) and SN (right half, blue) participants at visit 1 and visit 2; *p*-values are for comparison of PLWH and SN participants at each visit by t-tests. A total of 200 matched PLWH/SN pairs were evaluated. It should be noted that each of the acceleration measures (*y*-axes) reported in this figure was calculated as an age-acceleration residual, as described in *Methods*.

Epigenetic age acceleration, as measured by epigenetic clocks, was significantly higher in PLWH than in matched SN participants at visit 1 in all measures except grim epigenetic age acceleration (Figures 2A–D). This significant group difference persisted after the initiation of HAART at visit 2 in AAR, EEAA, and PEAA (all *p* < 0.001), although the magnitude of the difference was the same (difference in medians 6.6 years for AAR) or decreased (differences in medians of 9.1 and 7.7 years for EEAA and PEAA, respectively). Similarly, aaDNAmTL was significantly shorter (indicative of older epigenetic age) in PLWH at visits 1 and 2 than in the matched seronegative controls (Figure 2E; both *p* < 0.001), and the magnitude of the difference in the median aaDNAmTL between PLWH and SN participants decreased after the initiation of HAART, from 0.53 relative units to 0.39 relative units, reflecting a 26% change in the difference between groups from visit 1 to visit 2.

**FIGURE 3:**
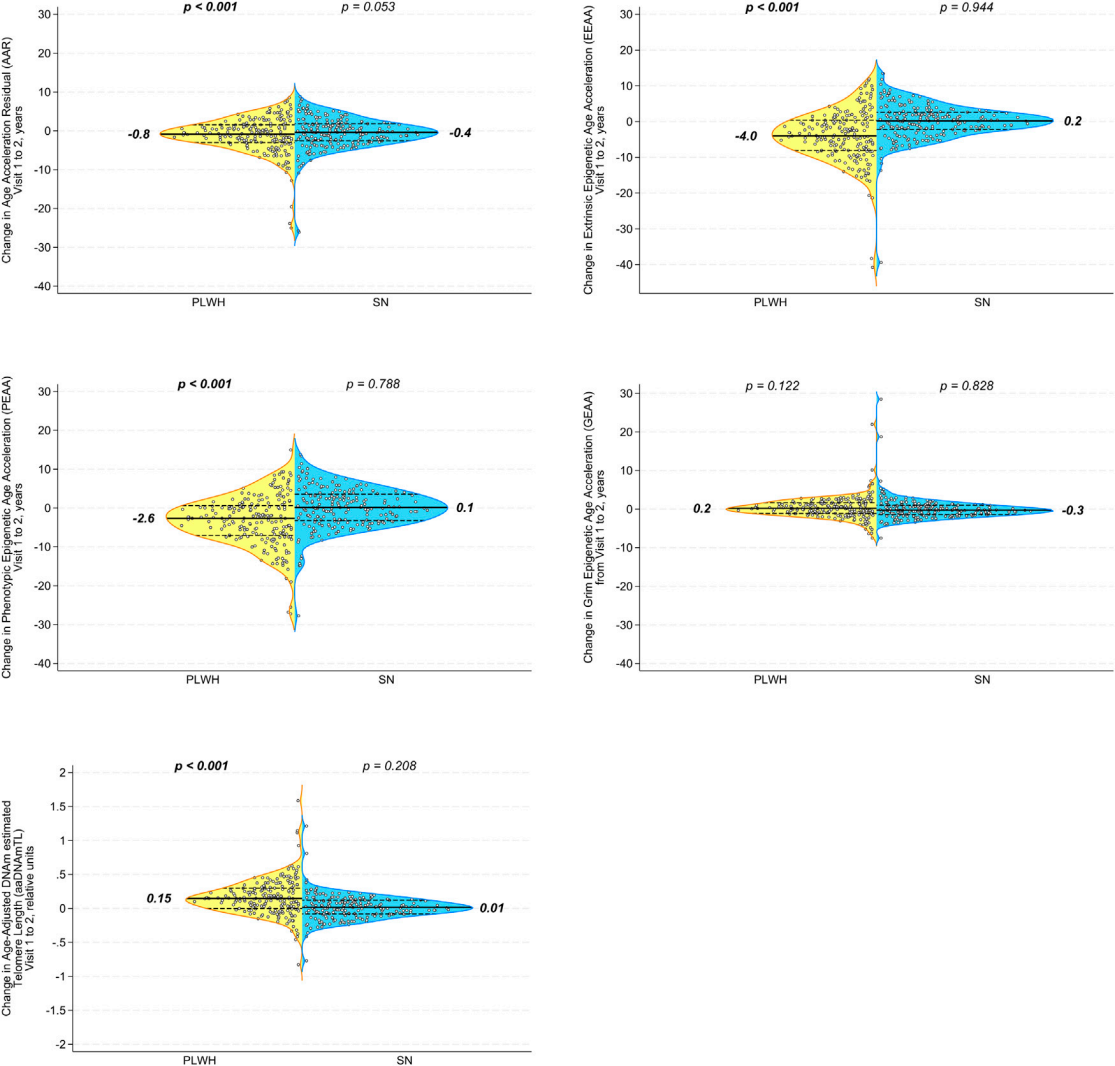
Initiation of HAART is associated with a significant decrease in acceleration in multiple epigenetic measures of aging in PLWH, with no significant changes over the same interval in the degree of accelerated aging in matched SN men. Violin plots of PLWH (n = 200 samples each visit, yellow) and HIV-SN (n = 200 samples each visit, blue) participants show the change in epigenetic age acceleration from pre-HAART or equivalent visit (visit 1) to the post-HAART or equivalent visit (visit 2) within each participant as measured by **(A)** AAR, **(B)** EEAA, **(C)** PEAA, **(D)** GEAA, and **(E)** aaDNAmTL. *p*-values are for comparison of changes to zero. It should be noted that each of the acceleration measures (*y*-axes) reported in this figure was calculated as an age-acceleration residual, as described in *Methods*.

When we examined the changes in epigenetic age acceleration within each individual between visits 1 and 2, we found that PLWH showed significant decreases from pre- to post-HAART in AAR, EEAA, and PEAA (Figures 3A–C; all *p* < 0.001) but not in GEAA (Figure 3D). A significant increase (indicative of longer telomeres/younger epigenetic age) was observed in intra-individual aaDNAmTL in PLWH from pre- to post-HAART visits (Figure 3E; *p* < 0.001). As hypothesized, no significant changes were observed in epigenetic age acceleration by any of the clock measures, or of aaDNAmTL, for matched SN participants between visits 1 and 2.

### Cumulative HIV exposure in PLWH since HAART initiation or initial HIV infection is associated with the degree of post-HAART acceleration in epigenetic aging

In light of the results that showed that PLWH still showed multiple significant differences from matched SN participants after HAART initiation, even after some improvement in most measures of epigenetic age acceleration, we explored whether this was related to the degree of HIV exposure over time in PLWH. Linear regression analyses were performed to examine whether the degree of viral exposure since initiating HAART (cumulative viral load from the estimated date of HAART initiation, as defined in *Methods*, to visit 2, CVL-H) was associated with the amount of epigenetic age acceleration still remaining after HAART initiation (visit 2) in each of the clocks and aaDNAmTL (Table 2). Higher log_10_ CVL-H was significantly associated with greater acceleration in EEAA and PEAA at visit 2 (*p* = 0.005). For every log10 increase in CVL-H, EEAA and PEAA increased by 2.0 and 1.8 years, respectively. No association was observed between CVL-H and AAR or GEAA at visit 2. Higher log10 CVL-H was associated with accelerated shortening of the estimated telomere length at visit two (*p* = 0.003). For every log10 increase in CVL-H, aaDNAmTL shortened by 0.08 relative units. We further examined whether the degree of lifetime viral exposure, i.e., since the estimated date of HIV infection (cumulative viral load from the estimated date of infection, as defined in *Methods*, to visit 2, CVL-I), was associated with the amount of epigenetic age acceleration observed at visit 2 in each clock and aaDNAmTL (Table 2). Higher log_10_ CVL-I was significantly associated with higher acceleration in EEAA at visit 2 (*p* = 0.023). For every log10 increase in CVL-I, EEAA increased by 2.1 years. No association was observed between CVL-I and AAR, PEAA, or GEAA at visit 2. Higher log10 CVL-I was associated with accelerated shortening of the estimated telomere length at visit 2 (*p* < 0.001). For every log10 increase in CVL-I, aaDNAmTL shortened by 0.11 relative units.

**TABLE 2:** Association of epigenetic age acceleration measures post-HAART (visit 2) in PLWH with cumulative HIV viral load since HAART initiation (CVL-H), and with cumulative HIV viral load since initial HIV infection (CVL-I).

	Linear regression β (*p-*value)^a^				
	AAR	EEAA	PEAA	GEAA	aaDNAmTL
**Log10 CVL-H (n = 200)**	0.27 (*0.59*)	2.0 (***0.005***)	1.8 (***0.005***)	0.44 (*0.25*)	−0.08 (***0.003***)
**Log10 CVL-I (n = 200)**	0.60 (*0.35*)	2.1 (***0.023***)	1.3 (*0.11*)	0.17 (*0.73*)	−0.11 (***<0.001***)

AAR, age acceleration residual; EEAA, extrinsic epigenetic age acceleration; PEAA, phenotypic epigenetic age acceleration; GEAA, grim epigenetic age acceleration; aaDNAmTL, age-adjusted DNA methylation-based estimate of telomere length; log10 CVL-H, log10-transformed viremia-copy years for cumulative HIV viral load from the estimated date of HAART initiation to post-HAART visit (visit 2); log10 CVL-I, log10-transformed viremia-copy years for cumulative HIV viral load from estimated date of HIV infection to post-HAART visit (visit 2).

a: Linear regression estimates: beta coefficients, and *p*-values (*p*-values bold if < 0.05) from simple linear regressions between log10-transformed cumulative viral loads (CVLs) and epigenetic measures at the post-HAART visit (visit 2). A separate regression was performed for each epigenetic clock on each of the two CVL measures (CVL-H or CVL-I); here, the CVL measure is the independent variable.

### Epigenetic age acceleration in PLWH according to the clinical response to HAART initiation

Given our observation that greater viral exposure since the initiation of HAART was related to greater epigenetic age acceleration in multiple measures, we explored whether PLWH who responded well to HAART differed from those who did not respond as effectively. We stratified PLWH into 2 groups based on the attainment of viral load suppression at visit 2: undetected (defined as those with HIV VL ≤ 50 copies per ml at visit 2, 2–3 years following the initiation of HAART, N = 125) and detected (defined as those with HIV VL > 50 copies per ml, N = 74). Table 3 shows the characteristics of these two PLWH groups, while Table 4 reveals the differences in the degree of epigenetic age acceleration, for each measure at each visit, between the detected PLWH and their matched SN controls and the undetected PLWH and their matched SN controls (as indicated by p_1_). At visit 1 (pre-HAART), epigenetic age acceleration was significantly higher in all epigenetic measures in the detected group and all measures except GEAA in the undetected group when compared with matched SN controls at visit 1. Similarly, at visit 2 (post-HAART initiation), epigenetic age acceleration was significantly higher in both the detected and undetected groups than in their respective matched SN participants in all measures except GEAA. When comparing epigenetic age acceleration in the undetected and detected groups (Table 4, indicated by p_2_), we found that aaDNAmTL was significantly shorter in the detected group at both visits 1 and 2, and EEAA and PEAA were significantly higher at visit 2 in the detected group than in the undetected group.

**TABLE 3:** Characteristics of PLWH stratified by undetected ( ≤ 50 copies/mL) or detected (>50 copies/mL) HIV plasma viral load (VL) at the post-HAART visit (visit 2).

	Undetected VL at post-HAART visit^a^		Detected VL at post-HAART visit^b^	*p*-value^c^
**Non-white race, n (%)**	17 (13.6)		11 (14.9)	0.97
**Hispanic ethnicity, n (%)**	6 (4.8)		10 (13.5)	0.055
**≥1 year college education, n (%)**	115 (92.0)		64 (86.5)	0.31
**Visit 1 to visit 2, years, mean (SD)**	2.9 (0.3)		2.8 (0.5)	0.04

At each visit	Undetected		Detected	
	Visit 1	Visit 2	Visit 1	Visit 2
**Age, years, mean (SD), [range]**	44.7 (8.4), [24.1–72.4]	47.6 (8.4), [27.0–75.5]	42.9 (6.6), [28.4–62.2]	45.7 (6.6), [31.4–65.2]
**Hepatitis C virus RNA-positive, n (%)**	5 (4.0)	7 (5.6)	3 (4.1)	3 (4.1)
**Hepatitis B virus surface antigen-positive, n (%)**	4 (3.2)	5 (4.0), n = 124	0 (0), n = 73	0 (0)
**Body mass index, kg/m** ^**2**^ **, mean (SD)**	25.4 (4.0), n = 118	25.2 (3.8), n = 112	25.9 (4.2), n = 68	25.8 (3.7), n = 73
**Smoking, cumulative pack years, mean (SD)**	15.1 (21), n = 121	15.7 (21), n = 123	11.3 (18)	12.1 (19), n = 73
**Absolute CD4 T-cell count, cells/mm** ^**3**^ **, mean (SD)**	442 (240), n = 124	611 (283), n = 124	367 (221), n = 73	461 (243)
**Plasma HIV viral load, copies/mL, mean (SD), median [range]**	63,498 (147,245)	49 (5)	111,339 (247,463)	25,310 (474,976)
	20,307 [50–948,000], n = 120	50 [20–50]	27,428 [50–1,733,258], n = 68	3,676 [51–775,439]
**Estimated time since HIV infection, years, mean (SD)**	9.5 (4.5), n = 108	12.4 (4.5), n = 108	8.9 (3.9), n = 65	11.7 (4.1), n = 65

a: n = 125 for the undetected PLWH group, unless otherwise indicated.

b: n = 74 for the detected PLWH group, unless otherwise indicated; one PLWH missing HIV VL at visit 2 could not be assigned to a group and was excluded from these analyses.

c: *p*-values from Student’s t-tests for continuous variables and chi-squared tests for count data (p-values bold if <0.05).

**TABLE 4:** Epigenetic age acceleration comparing PLWH with detected (VL > 50) and PLWH with undetected (VL ≤ 50) copies/mL to matched SN participants, and the two PLWH groups to each other, at each visit.

	Visit 1 (pre-HAART)		Visit 2 (post-HAART)	
	Difference in the PLWH group mean from the matched SN group, years or relative units (p_1_)^a^	Mean in the PLWH group, years or relative units (p_2_)^b^	Difference in the PLWH group mean from the matched SN group, years or relative units (p_1_)^a^	Mean in the PLWH group, years or relative units (p_2_)^b^
Undetected PLWH^c^				
AAR	6.2 (**<0.001**)	3.4	5.4 (**<0.001**)	2.1
EEAA	10.9 (**<0.001**)	6.2	6.4 (**<0.001**)	1.7
PEAA	9.0 (**<0.001**)	5.1	5.2 (**<0.001**)	1.1
GEAA	0.0 (0.933)	−0.1	0.5 (0.408)	0.2
aaDNAmTL	−0.502 (**<0.001**)	−0.272	−0.342 (**<0.001**)	−0.103
Detected PLWH^d^				
AAR	6.9 (**<0.001**)	4.2 (0.375)	6.9 (**<0.001**)	3.3 (0.205)
EEAA	14.0 (**<0.001**)	8.4 (0.075)	10.6 (**<0.001**)	5.2 (**0.009**)
PEAA	12.2 (**<0.001**)	7.2 (0.053)	9.8 (**<0.001**)	5.2 (**0.001**)
GEAA	1.5 (**0.018**)	0.8 (0.138)	1.5 (0.56)	1.1 (0.249)
aaDNAmTL	−0.598 (**<0.001**)	−0.387 (**0.012**)	−0.473 (**<0.001**)	−0.232 (**0.007**)

a: Difference between mean epigenetic values at indicated visit of the PLWH group and matched SN controls (PLWH group mean–SN group mean) in years (AAR, EEAA, PEAA, and GEAA) or relative units (aaDNAmTL); p_1_ is the *p*-value from the *t*-test comparing the PLWH group mean to the matched SN group mean (p-values bold if <0.05).

b: Mean epigenetic values at visit of the PLWH group in years (AAR, EEAA, PEAA, and GEAA) or relative units (aaDNAmTL); p_2_ is the *p*-value from the *t*-test comparing the detected PLWH mean to the undetected PLWH mean (p-values bold if <0.05).

c: PLWH stratified based on the undetected HIV VL at the post-HAART visit (< or = 50 copies/mL; n = 125).

d: PLWH stratified based on the detected HIV VL at the post-HAART visit (>50 copies/mL; n = 74).

We also examined the relationship between the lifetime cumulative viral load and epigenetic measures in the undetected and detected PLWH groups. At visit 1, in the undetected group that would become virally suppressed at visit 2, greater lifetime HIV exposure to date, i.e., higher log_10_ cumulative viral load since the estimated date of HIV infection (CVL-I), is correlated with higher EEAA (r = 0.2, *p* = 0.018) and PEAA (r = 0.21, *p* = 0.020) and shorter aaDNAmTL (r = −0.37, *p* < 0.001).

At visit 1 in the detected group that would retain a measurable viral load post-HAART, higher log_10_ CVL-I is correlated with higher AAR (r = 0.25, *p* = 0.04) and EEAA (r = 0.33, *p* = 0.005) and shorter aaDNAmTL (r = −0.33, *p* = 0.005). At visit 2, in the undetected group, only one significant correlation with log10 CVL-I persisted [shorter aaDNAmTL (r = −0.29, *p* = 0.0009)], and no significant correlations remained in the detected group.

### Demographic and behavioral factors associated with epigenetic aging acceleration pre- and post-HAART

Using mixed-effects model analyses accounting for all participants at both visits, we found that the HIV group (PLWH vs. SN participants) and study visit (visit 1 vs. visit 2) each maintained their significant association with AAR, EEAA, and PEAA (all *p* < 0.001) after adjusting for demographic and behavioral variables, including race, HBV status, tobacco smoking (cumulative pack years), and BMI (see Table 5). While higher tobacco smoking (cumulative pack years) was associated with a higher degree of PEAA (*p* = 0.027) in mixed-effects models, no other variables were significantly associated with epigenetic age acceleration in any of the clocks examined. Likewise, the study visit and HIV group each remained significantly associated with aaDNAmTL in models accounting for demographic and behavioral variables (both *p* < 0.001). White race was significantly associated with shorter methylation-based estimates of telomere length (*p* < 0.001) as has been demonstrated in previous studies of actual telomere length (Hunt et al., 2014). The interaction term study visit * HIV group was significantly associated with the degree of age acceleration in the EEAA and PEAA clocks (*p* < 0.001), demonstrating that the initiation of HAART in PLWH is significantly associated with a reduction in the degree of age acceleration observed in PLWH, in models adjusted for demographic and behavioral factors. Likewise, the study visit * HIV group interaction term was significantly associated with aaDNAmTL in models adjusted for demographic and behavioral factors (*p* < 0.001), suggesting a role of HAART in PLWH in mitigating the accelerated shortening of the estimated telomere length associated with HIV infection.

**TABLE 5:** Demographic variables associated with epigenetic age acceleration in mixed models.

Potential contributors to epigenetic age acceleration	F-value (*p*-value)^a^			
	AAR	EEAA	PEAA	aaDNAmTL
**Study visit**	17.0 (**<0.001**)	42.2 (**<0.001**)	22.1 (**<0.001**)	55.0 (**<0.001**)
**Visit 1 vs. visit 2**				
**HIV group, PLWH vs. SN participants**	125.0 (**<0.001**)	147.0 (**<0.001**)	134.0 (**<0.001**)	192.0 (**<0.001**)
Study visit*	1.40 (0.24)	35.1 (**<0.001**)	21.5 (**<0.001**)	34.1 (**<0.001**)
**HIV group**				
**Race, non-white vs. white**	0.5 (0.50)	1.7 (0.20)	0.3 (0.58)	29.4 (**<0.001**)
**Hepatitis B status, HBsAg– vs. +**	3.3 (0.069)	0.25 (0.62)	3.1 (0.077)	0.07 (0.80)
**Tobacco smoking, cumulative pack years**	3I73 (0.054)	1.47 (0.22)	4.9 (0.027)	1.6 (0.20)
**Body mass index, kg/m** ^**2**^	1.1 (0.29)	0.6 (0.44)	0.2 (0.68)	0.3 (0.57)

AAR, age acceleration residual; EEAA, extrinsic epigenetic age acceleration; PEAA, phenotypic epigenetic age acceleration; aaDNAmTL, age-adjusted DNA methylation-based estimate of telomere length; PLWH, persons living with HIV; SN = HIV seronegative controls; HBsAg, hepatitis B surface antigen.

a: F-values and Pr > F *p*-values (*p*-values bold if < 0.05) from mixed models taking all variables into account in all participants at both visits for each epigenetic measure.

### T-cell subsets are associated with the acceleration of epigenetic aging

As noted in Table 1, the mean absolute CD4 T-cell counts were significantly reduced at both visits 1 and 2 for PLWH compared with matched SN participants. In addition, the mean absolute CD4 T-cell counts increased significantly from visit 1 to visit 2 in PLWH. These differences in the absolute CD4 T-cell counts between study groups and visits indicated the need to develop models adjusted for differences and changes in immune cell composition when examining associations with epigenetic age acceleration. Using the absolute counts of total CD4 and CD8 T cells, and of naïve, activated, and senescent CD4 and CD8 T-cell subsets, we identified the consensus best-fit model across the epigenetic measures (as described in *Methods*), which contained the total CD4 and CD8 T cells, plus naive, activated, and senescent CD8 T-cell subsets. The same process using proportions of T cells yielded an identical consensus model. Using absolute T-cell counts, we found significant associations between epigenetic age acceleration (AAR, EEAA, and PEAA) and CD4 T cells, CD8 T cells, and naïve CD8 T cells (Table 6). In addition, there was a significant association between senescent CD8 T cells and AAR and EEAA. Finally, aaDNAmTL was associated with total, naïve, and senescent CD8 T-cell counts. Importantly, when these cell count adjustments were made, the association with study visit and the study visit*HIV group interaction were no longer significantly associated with epigenetic age acceleration in any of the epigenetic clocks or aaDNAmTL. Similar results were obtained with the same consensus model when using proportions of T cells (data not shown).

**TABLE 6:** T-cell subsets associated with epigenetic age acceleration.

Potential contributors to epigenetic age acceleration	F-value (*p*-value)^a^			
	AAR	EEAA	PEAA	aaDNAmTL
**Study visit**	0.06 (0.81)	0.97 (0.32)	0.63 (0.43)	0.48 (0.49)
**Visit 1 vs. visit 2**				
**HIV group**	3.01 (0.083)	3.2 (0.076)	10.9 (**0.001**)	3.30 (0.070)
**PLWH vs. SN participants**				
Study visit*	0.01 (0.93)	0.54 (0.46)	0.33 (0.56)	1.68 (0.20)
**HIV group**				
**Absolute CD4 T cell count, cells/mm** ^**3**^	4.20 (**0.042**)	24.5 (**<0.001**)	11.6 (**<0.001**)	28.4 (**<0.001**)
**Absolute CD8 T-cell count, cells/mm** ^**3**^	0.56 (0.45)	43.4 (**<0.001**)	12.4 (**<0.001**)	60.8 (**<0.001**)
**Absolute naïve** **(CD45RA^+^CCR7^+^)** **CD8 T-cell count, cells/mm** ^**3**^	1.03 (0.31)	166 (**<0.001**)	96.2 (**<0.001**)	226 (**<0.001**)
**Absolute activated** **(HLA-DR^+^CD38^+^)** **CD8 T-cell count, cells/mm** ^**3**^	4.52 (**0.0034**)	0.10 (0.75)	0.20 (0.65)	12.0 (**<0.001**)
**Absolute senescent** **(CD28^−^CD57^+^)**	0.90 (0.34)	15.5 (**<0.001**)	1.10 (0.29)	28.92 (**<0.001**)
**CD8 T-cell count, cells/mm** ^**3**^				

AAR, age acceleration residual; EEAA, extrinsic epigenetic age acceleration; PEAA, phenotypic epigenetic age acceleration; aaDNAmTL, age-adjusted DNA methylation-based estimate of telomere length; PLWH, persons living with HIV; SN, HIV seronegative controls.

a: F-values and Pr > F *p*-values (*p*-values bold if < 0.05) from best-fit flow cytometry mixed models using absolute T-cell counts as described in *Methods*, taking all variables into account in all participants at both visits for each epigenetic measure.

### Epigenome-wide methylation patterns associated with the initiation of HAART

WGCNA (Langfelder and Horvath, 2008) was used to identify clusters of CpG DNA methylation sites (CpGs) that are correlated with each other across all samples analyzed (co-methylation modules), using methylation levels measured at over 850,000 individual CpGs on the Infinium MethylationEPIC BeadChip. Sixty-seven co-methylation modules were identified, as previously described (Breen et al., 2022; Breen et al., 2023). Within SN participants, no modules had significantly different mean eigenvector methylation when comparing visit 1 to visit 2. Within PLWH, 11 modules showed statistically significant mean eigenvector methylation differences (*p* < 7.5 × 10^−4^ adjusting for multiple comparisons) between visits 1 and 2 (Table 7). Of the 11 modules identified, 9 of them (up to module 18) were previously identified as significantly different over the course of initial HIV infection (Breen et al., 2022; Breen et al., 2023) but changed in the opposite direction as those observed in these analyses pre/post-HAART initiation (Table 7, eigenvector change). This is consistent with our observations from some of the epigenetic clocks and aaDNAmTL, suggesting that HAART initiation partially reverses the methylation patterns observed as a result of HIV infection. For each CpG within each of the 11 modules significantly associated with the initiation of HAART, an intramodular connectivity measure (kME value) can be calculated, and kME values above the most stringent cutoff of 0.85 identify “hub” CpGs and potential genes of interest within each module (Horvath and Dong, 2008). All individual CpG sites within the two newly described modules associated with HAART initiation but not initial HIV infection (modules 19 and 20) are listed in Supplementary Table S6; CpG lists for the other nine modules associated with both HAART initiation and initial HIV infection are given in the study by Breen et al., and these nine modules are identical and unchanged from our previous work (Breen et al., 2022).

**TABLE 7:** Weighted gene co-methylation network analysis (WGCNA) modules (n = 11) significantly associated with the initiation of HAART in PLWH.

Co-methylation module^a^	# of CpGs in module	Module eigenvector methylation^c^				# (%) of CpGs with kME ≥ 0.85
		Visit 1 mean (SD)	Visit 2 mean (SD)	Eigenvector change^d^	*p*-value^e^	
**1**	133,037	−0.017 (0.027)	−0.004 (0.024)	**↑** 0.013	3.3 × 10^−6^	37,284 (28)
**4**	9,775	−0.018 (0.021)	−0.006 (0.024)	**↑** 0.012	3.5 × 10^−7^	1,596 (16)
**5**	5,615	0.022 (0.020)	0.013 (0.021)	**↓** 0.009	1.8 × 10^−5^	672 (12)
**6**	5,184	−0.012 (0.024)	−0.003 (0.025)	**↑** 0.009	6.8 × 10^−5^	892 (17)
**8**	1,677	−0.020 (0.019)	−0.011 (0.023)	**↑** 0.009	1.6 × 10^−5^	141 (8)
**9**	1,019	−0.018 (0.025)	−0.003 (0.024)	**↑** 0.015	2.6 × 10^−9^	50 (5)
**14** ^**b**^	106	0.021 (0.026)	0.011 (0.023)	**↓** 0.010	4.9 × 10^−4^	5 (4.7)
**15**	94	0.015 (0.025)	0.003 (0.024)	**↓** 0.012	2.6 × 10^−6^	1 (1)
**18** ^**b**^	38	−0.025 (0.035)	−0.007 (0.022)	**↑** 0.018	4.1 × 10^−8^	17 (45)
**19**	91	−0.012 (0.023)	−0.003 (0.023)	**↑** 0.009	2.3 × 10^−4^	22 (24)
**20**	71	−0.008 (0.024)	0.001 (0.025)	**↑** 0.009	6.2 × 10^−4^	2 (3)

a: Each co-methylation module (module numbers shown in bold) is a cluster of CpG methylation sites (CpGs) within the >850,000 sites evaluated on the Infinium MethylationEPIC BeadChip, identified by WGCNA, to be correlated with each other; any one CpG site belongs to only one module. Out of a total of 67 modules identified by WGCNA, identical to the modules reported by Breen et al. (2022), the 11 modules shown are those that are significantly associated with HAART initiation between visit 1 and visit 2 in the PLWH. Modules up to #18 are numbered as assigned by Breen et al. (2022). Modules #19 and 20 are numbered according to the number of CpGs (largest to smallest) contained in those two modules.

b: Modules containing CpGs that fall within pathways with significant adjusted *p*-values in the enrichment analysis.

c: Methylation levels defined as ratio of intensities, and module eigenvector defined as the first principal component in the module methylation matrix. Data are based on 200 PLWH with observations at both visits 1 and 2.

d: Direction (up arrow = positive or increased methylation; down arrow = negative or decreased methylation) and magnitude of change in the mean module methylation (visit 2 mean module eigenvector–visit 1 module eigenvector).

e: Results from a non-parametric group comparison test (Kruskal–Wallis) comparing the mean module eigenvector methylation from visits 1 to 2 over the course of HAART initiation (PLWH only); the level of significance adjusting for multiple comparisons was *p* < 0.05/67 or <7.5 × 10^−4^. No significant differences were observed in the mean module eigen methylation from visits 1 to 2 for any module in SN controls.

Enrichment analyses were performed using the Enrichr gene list enhancement tool (Supplementary Table S7, modules 19 and 20; enrichment analyses results for the other 9 modules can be found in the study by Breen et al. (2022)). Pathway analyses of the two newly identified modules 19 and 20 revealed that each of these modules contained CpGs from large numbers of pathways, including cell–cell adhesion, regulation of cytokines and Toll-like receptors (module 19), and morphogenesis, neuropeptide signaling, and DNA de-methylation (module 20). Although our enrichment analysis does not identify direct functional or mechanistic connections between methylation levels of the CpGs within each module, we use this analysis to suggest potential functional themes represented in or by each CpG module.

## Discussion

PLWH are at increased risk for the early onset of frailty (Desquilbet et al., 2007) and age-related illnesses, including hypertension, cardiovascular disease, kidney failure, type 2 diabetes mellitus, and bone fracture (Guaraldi et al., 2011; Bhatia et al., 2012). The increased prevalence of frailty and age-related illnesses even in the setting of successful viral suppression with HAART (Bhatia et al., 2012) raises the question of what the underlying mechanism of the accelerated aging observed in HIV infection is.

In this large longitudinal study, we demonstrate that PLWH are persistently epigenetically older than age-matched controls even after the initiation of HAART, although the magnitude of the difference decreases with HAART. A significant within-person decrease is observed in the degree of acceleration in PLWH between pre- and post-HAART visits, indicating that the accelerated aging that occurs during infection is partially mitigated by treatment. We further demonstrate that the cumulative viral load is associated with the degree of acceleration, and that even in PLWH who demonstrate clinical treatment success (i.e., undetectable HIV VL after HAART initiation), epigenetic acceleration persists compared to matched SN controls. Our study is the first to demonstrate that T-cell subset changes are associated with the observed decrease in epigenetic age accelerations across multiple epigenetic measures (epigenetic clocks and estimates of TL) in PLWH.

Our findings are consistent with those of previous studies examining the effects of HAART on epigenetic age acceleration in HIV infection. In a much smaller pilot study in 2020 (n = 15 PLWH/control pairs), we reported that AAR, EEAA, PEAA, and GEAA were all significantly higher in PLWH than that in age-matched control participants, and that 18–24 months after HAART initiation, PEAA and GEAA were no longer significantly different between cases and controls, while AAR and PEAA remained significantly higher in PLWH, although the magnitude of the difference decreased (Wang et al., 2018). A conflicting finding of this study of 200 PLWH/SN pairs is that GEAA was not significantly higher in PLWH at pre- or post-treatment visits, and we have previously shown in a large longitudinal study that GEAA does not significantly change with HIV seroconversion (Breen et al., 2022). In another study examining age acceleration in methylation patterns in the setting of treated HIV, methylation age was shown to be accelerated by 4.9 years in treated HIV infection (Gross et al., 2016), which is consistent with the 5, 6.6, and 7 years of acceleration we found in AAR, EEAA, and PEAA, respectively. In a recent study examining the effects of age, antiretroviral therapy, and a cancer diagnosis on epigenetic aging in HIV infection, age acceleration in PLWH, as measured by Horvath’s AAR, was mitigated by antiretroviral therapy and not further accelerated by a diagnosis of cancer (Qing et al., 2023).

In a longitudinal study of 168 HAART-naïve participants with HIV pre- and 2 years post-HAART and 44 participants without HIV with similar age and sex distribution, AAR, GEAA, and PEAA were all significantly higher in cases than controls prior to the initiation of therapy, and the degree of epigenetic aging was more pronounced in participants with CD4 counts less than 200 cell/uL (Esteban-Cantos et al., 2021). After 2 years of antiretroviral therapy, PEAA remained significantly higher in cases (Esteban-Cantos et al., 2021), which is consistent with our study. In contrast to our results, in the study conducted by Esteban-Cantos et al. (2021), GEAA was significantly higher pre- and post-treatment (Esteban-Cantos et al., 2021). Finally, using methylation-based estimates of leukocyte subsets, PLWH demonstrated more differentiated T-cell phenotypes and pro-inflammatory leukocytes (Esteban-Cantos et al., 2021). A more recent longitudinal study following 44 long-term aviremic PLWH (HIV VL < 50 copies/mL for at least 1 year prior to recruitment) over 4 years demonstrated that AAR decreased and PEAA and GEAA did not change after 4 years of successful antiretroviral therapy (Esteban-Cantos et al., 2022). Higher levels of AAR, PEAA, and GEAA measures at the baseline were associated with serious clinical events (Esteban-Cantos et al., 2022). Most recently, a study of 81 participants in the Swiss HIV Cohort Study, with a median follow up of 8 years during untreated infection and 9.8 years on suppressive ART, revealed that epigenetic age acceleration increased during the period of untreated infection and diminished significantly during suppressive ART in several clocks examined, including AAR, Hannum, PEAA, and skin and blood age acceleration (Schoepf et al., 2023), which is consistent with our prior work (Sehl et al., 2021) and the current report.

A novel aspect of our study is the direct measurement of T-cell subsets via flow cytometry from the same PBMC samples and study visits where epigenetic age is measured. We demonstrate that T-cell composition changes are associated with changes in epigenetic clocks that occur with HAART. We previously directly demonstrated that T-cell composition changes, especially increases in activated and/or senescent T cells and decreases in naive T cells, are temporally associated with epigenetic accelerations that occur in the setting of initial HIV infection (Breen et al., 2022; Breen et al., 2023). Further studies are needed to determine if changes in these specialized T-cell subsets are causal, i.e., driving the degree of epigenetic acceleration in HIV infection even during what is considered a clinically successful treatment.

Another unique and important finding of our study over the course of HAART initiation is the mitigation, but not normalization, of the degree of the estimated accelerated telomere loss as measured by aaDNAmTL, a measure of cell replicative history. As this measure is associated with important health outcomes, such as time to cancer diagnosis and risk of mortality (Lu et al., 2019a; Lu et al., 2019b), we hypothesize that its relationship to HIV infection, even after the initiation of HAART, may be related to adverse health outcomes observed in this population. Further studies are in progress to examine whether residual accelerations after HAART initiation in DNAmTL, as well as the epigenetic clocks, are related to future risk of cancer, frailty, and mortality in PLWH.

In addition to the epigenetic clocks and estimate of TL examined in this study, we investigated global methylation patterns associated with HAART initiation, which we recently evaluated over the course of initial HIV infection. The majority of the modules we find associated with HAART were previously found to change during the process of HIV seroconversion (Breen et al., 2022; Breen et al., 2023), and as expected, the direction of the change in module eigenvector methylation after HAART is the reverse of that following HIV infection, suggesting that the accelerated biologic aging is reduced. Two modules not associated with HIV infection were identified in our pre/post-HAART analyses, one module with probes located in pathways involved in cytokine regulation, Toll-like receptor signaling, and morphogenesis, and the second module with probes located in pathways involved in chemotaxis, neuron synaptic transmission, and morphogenesis.

Although the MACS cohort is an extraordinary cohort and a resource of longitudinal biobanked samples with detailed clinical and demographic history, it does have some limitations. One such limitation of our study includes its focus on a study population that is composed of men only. In addition, most of our study participants were white and non-Hispanic. Similarly, the definition and nature of HAART have changed over the years. Further studies are needed to validate our findings in populations of women and men of diverse racial and ethnic backgrounds, with more modern HAART regimens.

We report persistent accelerated biological aging in PLWH after the initiation of treatment with HAART, with a significant mitigation of the degree of acceleration post-HAART. We find that the cumulative viral load is correlated with the degree of acceleration and that T-cell composition changes are associated with changes in accelerated aging. Further studies are needed, and are currently in progress, to examine whether the persistent accelerations observed in epigenetic measures over the first few years of HAART are sustained over extended periods of time on treatment, and if the persistently accelerated epigenetic measures are associated with longer-term adverse health outcomes in PLWH.

## Data Availability

The data analyzed in this study is subject to the following licenses/restrictions: The raw Infinium MethylationEPIC BeadChip methylation data that support the findings reported in this study cannot be deposited in a public repository at this time because of the policies of the MACS/WIHS Combined Cohort Study (MWCCS) from which they were generated. Per MWCCS policies, these raw data will be released via a concept sheet approval process (https://statepi.jhsph.edu/mwccs/work-with-us/) once the original aims of our approved study are complete. All analytic data utilized in this paper (calculated age-regressed epigenetic clock and estimated telomere length data, T cell counts and percentages, as well as necessary deidentified demographic or descriptive data), have been deposited with the MWCCS, and are available upon reasonable request via the MWCCS concept sheet approval process (https://statepi.jhsph.edu/mwccs/work-with-us/). All data related to this paper was obtained under MWCCS concept sheet C15039. Requests to access these datasets should be directed to https://statepi.jhsph.edu/mwccs/work-with-us/.
