# Supplementary material for: Decreased but persistent epigenetic age acceleration is associated with changes in T-cell subsets after initiation of highly active antiretroviral therapy in persons living with HIV
Source: Front Bioinform. 2024 May 24;4:1356509. doi: 10.3389/fbinf.2024.1356509 (PMC11157435; doi:10.3389/fbinf.2024.1356509)
Supplement: Supplementary file 2 [file Table1.pdf]

SUPPLEMENTARY TABLES

TABLE S1 Summary of antiretroviral medications taken by PLWH (n=200).

Type of therapy	Ever taken[a], n (%)	Currently taking[b], n (%)
Non nucleoside reverse transcriptase inhibitor	104 (52.0)	77 (38.5)
Protease inhibitor	156 (78.0)	131 (65.5)
Nucleoside reverse transcriptase inhibitor	198 (99.0)	185 (92.5)
Entry inhibitor	0 (0.0)	0 (0.0)
Integrase inhibitor	1 (0.5)	0 (0.0)

a: Ever taken = ever used, prior to Visit 2
b: Currently taking at Visit 2

TABLE S2 Mean absolute T cell counts of PLWH and SN at Visits 1 and 2.

	**Visit 1 (pre-HAART or equivalent) Mean (SD), n**			**Visit 2 (post-HAART or equivalent) Mean (SD), n**		
**T cell population**	**PLWH**	**SN**	**p-value[a]**	**PLWH**	**SN**	**p-value**
**CD4 T cells, cells/mm^3**	414 (235), n=198	997 (323), n=193	**<0.001**	555 (277), n=199	969 (299), n=196	**<0.001**
**CD8 T cells, cells/mm^3**	1113 (527), n=198	570 (265), n=193	**<0.001**	1017 (473), n=199	561 (259), n=196	**<0.001**
**Naive ($CD45RA^+CCR7^+$) CD4 T cells, cells/mm^3**	149 (122), n=193	362 (218), n=191	**<0.001**	219 (158), n=197	353 (210), n=195	**<0.001**
**Naive ($CD45RA^+CCR7^+$) CD8 T cells, cells/mm^3**	138 (106), n=193	191 (118), n=191	**<0.001**	204 (150), n=197	180 (111), n=195	0.083
**Activated ($HLA\text{-}DR^+CD38^+$) CD4 T cells, cells/mm^3**	29 (16), n=193	26 (13), n=190	**<0.001**	23 (12), n=197	25 (11), n=193	0.070
**Activated ($HLA\text{-}DR^+CD38^+$) CD8 T cells, cells/mm^3**	224 (199), n=193	19 (15), n=190	**<0.001**	86 (86), n=197	20 (14), n=193	**<0.001**
**Senescent ($CD28^-CD57^+$) CD4 T cells, cells/mm^3**	37 (40), n=193	35 (47), n=191	**<0.001**	41 (51), n=197	33 (42), n=195	**<0.001**
**Senescent ($CD28^-CD57^+$) CD8 T cells, cells/mm^3**	223 (183), n=193	103 (114), n=191	**<0.001**	215 (156), n=197	103 (111), n=195	**<0.001**

a: p-values are for comparison of PLWH vs. SN at each visit by t-tests (p-values bold if < 0.05)

TABLE S3 Mean percentages of total T cells and T cell subsets within the live lymphocyte population of PLWH and SN at Visits 1 and 2.

	Visit 1, Mean % of live lymphocytes (SD)			Visit 2, Mean % of live lymphocytes (SD)		
**T cell population**	**PLWH (n=178)**	**SN (n=177)**	**p-value[a]**	**PLWH (n=177)**	**SN (n=170)**	**p-value**
**Total T cells**	67.4% (0.11)	65.6% (0.11)	0.129	65.8% (0.11)	64.6% (0.11)	0.306
**CD4 T cells**	29.0% (0.14)	63.8% (0.09)	**<0.001**	38.3% (0.15)	64.8% (0.10)	**<0.001**
**CD8 T cells**	63.7% (0.13)	30.9% (0.09)	**<0.001**	55.3% (0.14)	30.00% (0.08)	**<0.001**
**Naive ($CD45RA^+CCR7^+$) CD4 T cells**	7.2% (0.06)	15.2% (0.08)	**<0.001**	10.2% (0.07)	15.1% (0.08)	**<0.001**
**Naive ($CD45RA^+CCR7^+$) CD8 T cells**	5.2% (0.04)	6.8% (0.04)	**<0.001**	7.1% (0.04)	6.4% (0.04)	0.082
**Activated ($HLA\text{-}DR^+CD38^+$) CD4 T cells**	1.3% (0.01)	1.1% (<0.01)	**<0.001**	1.0% (<0.01)	1.1% (<0.01)	0.159
**Activated ($HLA\text{-}DR^+CD38^+$) CD8 T cells**	8.7% (0.06)	0.74% (0.01)	**<0.001**	3.1% (0.03)	0.7% (0.01)	**<0.001**
**Senescent ($CD28^-CD57^+$) CD4 T cells**	1.5% (0.02)	1.4% (0.02)	0.427	1.6% (0.02)	1.4% (0.02)	0.325
**Senescent ($CD28^-CD57^+$) CD8 T cells**	8.5% (0.06)	3.8% (0.04)	**<0.001**	7.8% (0.05)	3.6% (0.04)	**<0.001**

a: p-values are for comparison of PLWH vs. SN at each visit by t-tests (p-values bold if <0.05)

TABLE S4. Within-person change in absolute T cell counts of PLWH and SN, Visit 2 - Visit 1.

	**Within-person change in absolute T cell counts, Visit 2 - Visit 1**					
	**PLWH**			**SN**		
**T cell population**	**Mean (SD), n**	**Median (IQR), n**	**p-value[a]**	**Mean (SD), n**	**Median (IQR), n**	**p-value[a]**
**CD4 T cells, cells/mm^3**	144 (244), n=197	131 (237), n=197	**<0.001**	-21 (246), n=190	-19 (255), n=190	0.23
**CD8 T cells, cells/mm^3**	-89 (493), n=197	-77 (510), n=197	**0.012**	-11 (196), n=190	-15 (160), n=190	0.44
**Naive (CD45RA$^+$CCR7$^+$) CD4 T cells, cells/mm^3**	74 (119), n=191	57 (129), n=191	**<0.001**	-9 (108), n=187	-5 (115), n=187	0.23
**Naive (CD45RA$^+$CCR7$^+$) CD8 T cells, cells/mm^3**	71 (131), n=191	48 (110), n=191	**<0.001**	-9 (69), n=187	-6 (59), n=187	0.09
**Activated (HLA-DR$^+$CD38$^+$) CD4 T cells, cells/mm^3**	-6 (16), n=191	-3 (17), n=191	**<0.001**	-1 (9), n=185	-1 (10), n=185	0.050
**Activated (HLA-DR$^+$CD38$^+$) CD8 T cells, cells/mm^3**	-138 (177), n=191	-84 (164), n=191	**<0.001**	0 (14), n=185	0 (10), n=185	0.69
**Senescent (CD28$^-$CD57$^+$) CD4 T cells, cells/mm^3**	5 (32), n=191	0 (22), n=191	**0.031**	-3 (24), n=187	0 (11), n=187	0.12
**Senescent (CD28−CD57+) CD8 T cells, cells/mm^3**	-9 (113), n=191	-1 (113), n=191	0.27	-3 (75), n=187	-3 (36), n=187	0.64

a: p-values from t-test for change within each participant group for differences from zero (p-values bold if <0.05)

TABLE S5 Pairwise correlations of epigenetic clocks (AAR, EEAA, PEAA, GEAA), and age-adjusted estimated telomere length (aaDNAmTL) with each other, among PLWH and SN at Visit 1 (pre-HAART or equivalent) and Visit 2 (post-HAART or equivalent).

	**Pearson coefficients (p-values[a])**				
**Visit 1, PLWH**	**AAR**	**EEAA**	**PEAA**	**GEAA**	**aaDNAmTL**
**AAR**	1.00	0.72 (**<0.001**)	0.61 (**<0.001**)	0.15 (**0.034**)	-0.54 (**<0.001**)
**EEAA**		1.00	0.74 (**<0.001**)	0.14 (**0.048**)	-0.73 (**<0.001**)
**PEAA**			1.00	0.33 (**<0.001**)	-0.57 (**<0.001**)
**GEAA**				1.00	0.0041 (0.95)
**Visit 1, SN**	**AAR**	**EEAA**	**PEAA**	**GEAA**	**aaDNAmTL**
**AAR**	1.00	0.62 (**<0.001**)	0.59 (**<0.001**)	0.19 (**0.007**)	-0.52 (**<0.001**)
**EEAA**		1.00	0.73 (**<0.001**)	0.26 (**<0.001**)	-0.78 (**<0.001**)
**PEAA**			1.00	0.54 (**<0.001**)	-0.62 (**<0.001**)
**GEAA**				1.00	-0.12 (0.091)
**Visit 2, PLWH**	**AAR**	**EEAA**	**PEAA**	**GEAA**	**aaDNAmTL**
**AAR**	1.00	0.66 (**<0.001**)	0.56 (**<0.001**)	0.047 (0.51)	-0.55 (**<0.001**)
**EEAA**		1.00	0.73 (**<0.001**)	0.26 (**<0.001**)	-0.77 (**<0.001**)
**PEAA**			1.00	0.42 (**<0.001**)	-0.68 (**<0.001**)
**GEAA**				1.00	-0.15 (**0.034**)
**Visit 2, SN**	**AAR**	**EEAA**	**PEAA**	**GEAA**	**aaDNAmTL**
**AAR**	1.00	0.50 (**<0.001**)	0.45 (**<0.001**)	-0.024 (**0.74**)	-0.46 (**<0.001**)
**EEAA**		1.00	0.65 (**<0.001)**	0.28 (**<0.001**)	-0.71 (**<0.001**)
**PEAA**			1.00	-0.46 (**<0.001**)	-0.53 (**<0.001**)
**GEAA**				1.00	-0.013 (0.86)

Abbreviations: AAR = Age-acceleration residual, EEAA = Extrinsic epigenetic age acceleration, PEAA = Phenotypic epigenetic age acceleration, GEAA = Grim epigenetic age acceleration, aaDNAmTL = age-adjusted DNA methylation-based estimate of telomere length, PLWH = persons living with HIV (n=200 samples at each visit), SN = seronegative controls (n=200 samples at each visit)

a: p-values for pairwise correlations (p-values bold if <0.05)

TABLE S6 (Excel File) Results from WGCNA, listing all CpGs in 2 previously unreported Modules, which are significantly associated with initiation of HAART in PLWH.

TABLE S7 (Excel file) Pathways enrichment analyses of genomic methylation data in 2 previously unreported WGCNA Modules associated with HAART initiation in PLWH.
